# Sprinting Biomechanics and Hamstring Injuries: Is There a Link? A Literature Review

**DOI:** 10.3390/sports9100141

**Published:** 2021-10-09

**Authors:** Rudy N. Kalema, Anthony G. Schache, Morgan D. Williams, Bryan Heiderscheit, Gabriel Siqueira Trajano, Anthony J. Shield

**Affiliations:** 1 Faculty of Health, School of Exercise and Nutrition Sciences, Queensland University of Technology, O Block Victoria Park Road, Kelvin Grove, QLD 4059, Australia; g.trajano@qut.edu.au (G.S.T.); aj.shield@qut.edu.au (A.J.S.); 2 La Trobe Sport and Exercise Medicine Research Centre, La Trobe University, Bundoora, VIC 3086, Australia; a.schache@latrobe.edu.au; 3 Faculty of Life Sciences and Education, University of South Wales, Pontypridd CF37 IDL, UK; morgan.williams@southwales.ac.uk; 4 Department of Orthopedics and Rehabilitation, University of Wisconsin-Madison, Madison, WI 53705, USA; heiderscheit@ortho.wisc.edu

**Keywords:** hamstrings, sprinting, injury, biomechanics, gait, retrospective, prospective

## Abstract

Hamstring strain injury (HSI) is a common and costly injury in many sports such as the various professional football codes. Most HSIs have been reported to occur during high intensity sprinting actions. This observation has led to the suggestion that a link between sprinting biomechanics and HSIs may exist. The aim of this literature review was to evaluate the available scientific evidence underpinning the potential link between sprinting biomechanics and HSIs. A structured search of the literature was completed followed by a risk of bias assessment. A total of eighteen studies were retrieved. Sixteen studies involved retrospective and/or prospective analyses, of which only three were judged to have a low risk of bias. Two other case studies captured data before and after an acute HSI. A range of biomechanical variables have been measured, including ground reaction forces, trunk and lower-limb joint angles, hip and knee joint moments and powers, hamstring muscle–tendon unit stretch, and surface electromyographic activity from various trunk and thigh muscles. Overall, current evidence was unable to provide a clear and nonconflicting perspective on the potential link between sprinting biomechanics and HSIs. Nevertheless, some interesting findings were revealed, which hopefully will stimulate future research on this topic.

## 1. Introduction

Hamstring strain injury (HSI) is the most prevalent noncontact muscle injury experienced in amateur and professional football codes [1,2,3,4]. This injury can be frustrating for the athlete and the treating clinician because training and/or matches are missed (usually for a minimum of two weeks) and the risk of recurrence upon return to play (RTP) is relatively high. HSIs not only affect the player’s health and psychosocial wellbeing, but can also adversely impact team performance [5] and football club finances [6,7]. Despite all the efforts performed to date by both researchers and clinicians to address the problem of HSIs, the incidence rate in elite sport remains unchanged [8,9].

**Figure 1:**
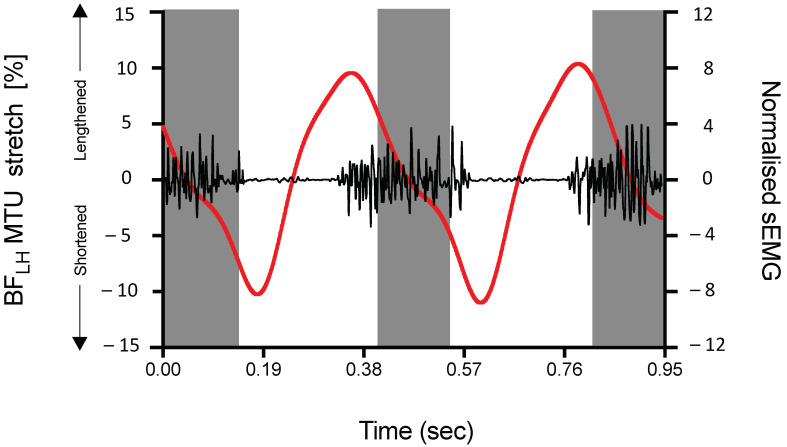
BF_LH_ MTU stretch and biceps femoris (BF) surface electromyographic (sEMG) activity during maximal sprinting. Experimental data obtained from Schache et al. [25]. The black line represents BF sEMG activity (high-pass filtered at 20 Hz). BF sEMG was normalised to the linear envelope ‘grand’ mean (i.e., the mean of all the valid periods of sEMG activity over the stride cycle). The red line represents the change in BF_LH_ MTU stretch which was calculated as a percentage of the MTU length assumed during a neutral upright stance pose. All data are recorded simultaneously from the same participant for two consecutive stride cycles during sprinting at a speed of 9.7 m/s. Stance phase is indicated by a vertical grey shaded bar.

Whilst HSIs can occur when undertaking a variety of functional activities, the most common mechanism of injury involves sprinting, either during the acceleration or the maximal velocity phases [1,10,11,12]. Sprinting is a challenging task for the hamstrings from both a biomechanical [13,14,15,16] and neuromuscular [17,18,19] perspective. The muscle–tendon unit (MTU) for the biarticular hamstrings undergoes an active stretch–shortening cycle during the late swing and stance phases of the sprinting stride cycle (Figure 1). During late swing, the hamstrings absorb kinetic energy and negative work is done, with the amount of negative work increasing exponentially with faster running [13,15]. The hamstrings then remain highly active throughout the stance phase where they actively contribute to the generation of the propulsive ground force impulse and thereby assist in accelerating the body forward [20,21,22,23]. Overall, the hamstrings must contract rapidly and forcefully on a repetitive basis during sprinting, and it is believed that such loading conditions may potentially make the hamstrings (especially the biceps femoris long head (BF_LH_)) susceptible to injury [24].

Because sprinting is a complex skill that appears to push the hamstrings to their limit, it may be considered a highly potent training stimulus. Progressive and regular exposure to sprinting has therefore been advocated as an important HSI prevention strategy [26,27,28,29,30,31]. However, is exposure to sprinting all that is required for injury prevention, or is an athlete’s sprinting biomechanics important too? It is conceivable that a link might exist between certain sprinting biomechanics variables and HSIs. Mann and Sprague raised this idea in the literature forty years ago when they related the magnitude of the hip joint moment at foot strike during sprinting to the incidence of HSIs [32,33]. Research formally testing this potential link has gradually increased since this time, but a detailed synopsis of all the available literature is yet to be completed. Is an athlete’s sprinting biomechanics altered following HSI? If so, how? In addition, do these alterations resolve over time? Such questions formed the basis of this review. Our overall aim was to evaluate the current evidence underpinning the potential link between sprinting biomechanics and HSIs. We considered the review to be warranted given that many in the sports medicine community have recommended that strategies to optimise sprinting biomechanics should be included in HSI rehabilitation and prevention programs [26,27,30,34].

## 2. Literature Search

A structured search of the available literature via MEDLINE, PubMed, EMBASE, SPORTdiscus, AMED, and the Cochrane Library was conducted from inception to July 2021. To be included, articles were required to be peer-reviewed, in full text, in English language, involve human participants and incorporate laboratory or field-based measurements of discrete biomechanical variables for running at a speed of at least a moderate intensity (i.e., >5.0 m/s). Biomechanical variables of interest concerned movement, force production and/or muscle activation. The keywords for the search are presented in Table 1. The reference lists of articles retrieved were also manually searched for any relevant articles that were not identified electronically. 

A total of 16 studies were retrieved, including 11 retrospective studies, two prospective studies, and three studies that had both retrospective and prospective components. We also considered the findings from two additional case studies that contained data captured before and after an acute HSI. 

## 3. Risk of Bias Assessment

Three assessors (RK, GT and MW) independently used a modified version of the Quality in Prognosis Studies (QUIPS) tool (see Appendix A) to assess the risk of bias for the 16 studies (i.e., the two case studies were excluded). This tool has been previously described [35] and has been utilised in recent HSI-related systematic reviews [36,37]. Two of the three reviewers evaluated each study. Discrepancies between authors were resolved by a third reviewer. The QUIPS tool has six potential bias domains (study participation; study attrition; prognostic factor measurement; outcome measurement; study confounding; statistical analysis and reporting) each consisting of three to five specific criteria for the opportunity of bias (see Appendix A). Criteria are given a score of either ‘yes’ or ‘no’. When >75% of responses within a particular domain were ‘yes’, then the risk of bias was considered low in that domain. A study was considered to a have an overall low risk of bias if: (1) five out of six domains were assessed as having low bias; and (2) low bias occurred for the outcome measurement domain. Studies were otherwise classified as having a high risk of bias.

Only three of the 16 studies had a low risk of bias (Table 2) [38,39,40]. These three studies involved cross-sectional analyses where sprinting biomechanics variables were compared between people with and without a history of HSI. The most common potential source of bias for the retrospective studies was ‘study confounding variables’ (domain 5: 93%) which was related to whether potential confounders were defined, identified and accounted for in the study design and analysis. The second most common potential source of bias identified was ‘prognostic factors measurement’ (domain 3: 64%) which assessed whether the risk of measurement bias related to how the prognostic factor was measured. The most common potential sources of bias for the prospective studies were ‘prognostic factors measurement’ (domain 3: 100%) and ‘study confounding variables’ (domain 5: 100%). 

## 4. Do HSIs Affect Sprinting Biomechanics?

The findings from studies that investigated whether people with a history of HSI have altered sprinting biomechanics are summarised in Section 4.1 and Section 4.2. Table 3 contains the results from five studies that used a within-participant between-limb design (i.e., previously injured limb vs. uninjured limb), whereas Table 4 contains the results from 11 studies that used a between-group design where people with a history of HSI are compared to a control cohort with no history of HSI. Two studies had both within-participant between-limb as well as between-group components, hence there were a total of 14 separate studies retrieved from the literature search that involved cross-sectional analyses. Sprinting biomechanics data were recorded under variety of testing conditions. Five studies utilised a treadmill (motorised or non-motorised) whereas nine studies involved over-ground sprinting (either in a laboratory or out in the field). Because these alternative testing conditions do not appear to affect sprinting biomechanics substantially [54,55], the various studies were not separated on this basis. The findings from studies that completed on-field measurements of sprinting biomechanics at various time points in athletes with a recent HSI are summarised in Section 4.3.

### 4.1. Studies Using a Within-Participant Design

A total of five studies were identified from the literature that used a within-participant design to investigate between-limb differences in sprinting biomechanics in people with a history of unilateral HSI. A range of variables were considered, including ground reaction forces (GRFs), sagittal plane pelvis and lower-limb joint angles, sagittal plane hip and knee joint moments and powers, hamstring (BF_LH_) MTU stretch, and hamstring surface electromyographic (sEMG) activity. Overall, three studies found evidence of between-limb differences [42,44,49], whereas two studies reported no differences [43,46].

Two studies investigated lower-limb joint angles and moments during sprinting. Lee et al. [42] found the previously injured limb to display significantly less hip flexion during late swing relative to the control limb, with no between-limb differences in knee kinematics evident at any stage of the stride cycle. More recently, Higashihara et al. [49] found the previously injured limb to display significantly less hip flexion during mid swing, but significantly more hip- and knee flexion during late swing, compared to the control limb. Alternative testing procedures might explain the differing results between these two studies. Higashihara et al. [49] investigated maximal sprinting (average speed of 9.39 ± 0.17 m/s), whereas Lee et al. [42] investigated submaximal sprinting (average speed of 7.7 ± 0.1 m/s). It is possible that between-limb differences in hip and knee joint angles may not be truly revealed at submaximal sprinting speeds. With respect to hip and knee joint moments during sprinting, neither Lee et al. [42] nor Higashihara et al. [49] found significant between-limb differences to exist. 

Two studies investigated between-limb differences in BF_LH_ MTU stretch as well as biceps femoris (BF) sEMG activity during sprinting with contrasting outcomes [43,49]. Silder et al. [43] found no difference in the profiles for BF_LH_ MTU stretch and BF sEMG activity across the entire stride cycle for the previously injured limb compared to the control limb, whereas Higashihara et al. [49] found the previously injured limb to display significantly decreased BF_LH_ MTU stretch and BF sEMG activity during late swing.

Finally, two studies investigated between-limb differences in GRFs during sprinting in football players [44,46]. Brughelli et al. [44] found the peak horizontal force to be 46% less for the previously injured limb compared to the control limb for submaximal sprinting on a non-motorised treadmill. In contrast, Barreira et al. [46] found no between-limb difference in the peak horizontal force when maximally sprinting on a non-motorised curved treadmill. These conflicting results could be attributable to a variety of factors, including differences in treadmill design and testing speed, potential variability in HSI severity (e.g., injured athletes had missed at least one week of training and/or competition in the study by Brughelli et al. [44], whereas missed time only had to exceed 48 h in the study by Barreira et al. [46]) as well as possible differences in the period of time between the athletes’ HSI and experimental data collection.

### 4.2. Studies Using a Between-Group Design

A total of eleven studies investigated differences in sprinting biomechanics between people with and without a history of HSI. A range of variables were evaluated, including GRFs, three-dimensional (3D) trunk, pelvis and lower-limb joint angles, sagittal plane lower-limb joint moments, as well as sEMG activity for the hamstrings along with other hip and trunk muscles. Overall, six studies reported differences in sprinting biomechanics variables of some sort [38,40,41,44,45,51], whereas five studies found no significant differences at all [39,46,47,48,50].

Four studies measured trunk and/or lower-limb kinematics during sprinting, with two of these studies reporting between-group differences. Iboshi et al. [41] tested 12 elite male sprinters performing a maximal-effort over-ground sprint from a crouch start position. The orientation of the lower-limb at foot strike in the sagittal plane was measured for the fifth step. Compared to the uninjured group, sprinters with a history of HSI had a lower-limb orientation that resulted in a larger horizontal distance between the toe and the location of the centre of gravity. Daly et al. [45] measured 3D pelvis and lower-limb kinematics for 17 hurlers whilst running on a treadmill at a submaximal speed of 5.6 m/s. Relative to uninjured controls, previously injured hurlers displayed significantly more anterior pelvic tilt and hip flexion asymmetry during late swing as well as significantly more knee axial rotation asymmetry during late swing and early stance. In contrast, two recent studies have not found between-group differences in sprinting kinematics. Schuermans et al. [47] measured 3D trunk, pelvis and lower-limb kinematics during over-ground sprinting for a cohort of soccer players and significant between-group differences were not evident for any variable. Haugen et al. [48] investigated interlimb asymmetry during sprinting in previously injured and non-injured high-level sprinters. They included 14 different kinematic variables related to interlimb asymmetry in their analysis, but none differed significantly between groups. 

Only one study measured lower-limb joint kinetics during sprinting. Iboshi et al. [41] found that sprinters with a history of HSI displayed a significantly greater peak hip extension moment during early stance compared to an uninjured group (196.9 ± 57.4 Nm vs. 111.6 ± 25.1 Nm). However, there was no difference in the magnitude of the hip extension and knee flexion moments during late swing.

Two studies examined between-group differences in hamstrings’ sEMG activity during submaximal and maximal sprinting, but different sEMG properties were evaluated [45,50]. Daly et al. [45] found hurlers with a history of HSI to display relatively reduced late swing BF sEMG activity during submaximal treadmill running when compared to an uninjured control group. More recently, Crow et al. [50] measured onset and offset times of sEMG activity for the gluteus maximus, the medial hamstrings (combination of semimembranosus and semitendinosus sEMG activity) and the BF for elite level Australian Rules football players during maximal over-ground sprinting. The temporal behaviour of the sEMG activity was not found to be significantly different between players with and without a history of HSI for any of the muscles evaluated.

Finally, six studies investigated differences in GRFs during sprinting [38,39,40,44,46,51]. Four studies reported significant differences between groups, although findings were not consistent across studies. Brughelli et al. [44] measured GRFs for Australian Rules football players sprinting on a non-motorised treadmill at a submaximal speed. Horizontal force was found to be reduced by ~32% for players with a recent history of HSI. Lord et al. [38] also tested a group of Australian Rules football players with and without a history of HSI. They measured GRFs using a non-motorised curved treadmill whilst players performed 10 repeated maximum-effort sprints of 6 s duration with a 24 s recovery period between each sprint. For players with a history of HSI, the mean horizontal force for the tenth sprint was 13% less than that for the first sprint, whereas the injury-free control group only displayed a 3% reduction. Ishøi et al. [40] tested soccer players with and without a history of HSI performing a repeated-sprint test, consisting of six 30 m maximal over-ground sprints with a 90 s recovery period between each sprint. No significant difference between groups was found when comparing horizontal force production for the first sprint or when comparing the change in horizontal force production across the six sprints. When taking into account data for all six sprints, Ishøi et al. [40] did find players with a history of HSI to display a higher mean maximal sprinting velocity and better mechanical effectiveness (i.e., lower rate of decline in ratio of forces with increasing speed). Finally, Edouard et al. [51] compared sprint acceleration mechanics between 60 soccer players with a history of HSI and 224 injury-free players. Baseline testing occurred at the start of the season. Players performed maximal 30 m sprints from a standing start on artificial turf. A radar gun system was used to measure the instantaneous sprint velocity. The data from the radar gun were then input into a computational model to estimate horizontal force production [56]. Key variables included predicted maximal horizontal force production at zero velocity and predicted maximal sprinting velocity where horizontal force can still be produced. They did not find any difference between groups with respect to predicted maximal horizontal force production, but they found predicted maximal sprinting velocity to be significantly greater for players with a history of HSI. 

In contrast to the above results, two other studies exploring horizontal force production during sprinting have not found differences between people with and without a history of HSI. Mendiguchia et al. [39] used the radar gun system to estimate horizontal force production during 50 m maximal over-ground sprints for 14 soccer players who had recently RTP (~2 months ago) and for 14 players without prior injury. Net horizontal force was found to be similar between the groups with values of 6.9 ± 0.8 N/Kg and 6.8 ± 0.6 N/Kg for recently injured and uninjured players, respectively. Barreiera et al. [46] tested soccer players with and without a history of HSI when sprinting on a non-motorised curved treadmill. They too found no difference in the horizontal GRF between groups. 

### 4.3. Evidence from Within-Participant Repeated Measures Analyses of HSI Cases

If measurable changes to sprinting biomechanics occur following a recent HSI, then it is important to understand whether these changes are self-limiting and naturally resolve over time or whether they persist. Three studies have investigated changes in sprinting biomechanics over time in athletes that have sustained a HSI [39,57,58]. Mendiguchia et al. [39] investigated sprinting biomechanics for 14 semi-professional soccer players who were recovering from a recent HSI. Forward velocity during sprinting was measured by a radar gun, which was then used to estimate horizontal force production. Two assessments were performed for the injured group, the first at the time of RTP and the second ~2 months after RTP. Net horizontal force for the injured group increased in magnitude from the first to the second assessment. Sprinting biomechanics variables for the injured group at the second assessment matched equivalent data recorded for an injury-free control group (Table 4). In a subsequent study, using the same sprint protocol and radar gun instrumentation, but involving a case study design, Mendiguchia et al. [57] recorded sprinting biomechanics for a professional soccer player 8 days prior to a HSI and 33 days following the injury. Maximal horizontal power and net horizontal force were both found to be reduced by ~20% at the post-injury assessment compared to the pre-injury assessment, despite the player having been cleared to RTP based on other criteria. The reduced horizontal force during sprinting at the time of RTP was thought to be attributable to a persisting impairment in hamstring function. Finally, Setuain et al. [58] investigated longitudinal changes in sprinting biomechanics in a semi-professional soccer player who suffered a HSI. They used an inertial sensor unit mounted on the lumbar spine to estimate horizontal and vertical GRFs during sprinting at three time points: (i) during the preseason (prior to the injury); (ii) at the time of RTP (after a midseason HSI); and (iii) at the end of the season. The decrease in the magnitude of the horizontal GRF with faster running at RTP (second time point) was more substantial for the injured limb compared to the unaffected (contralateral) limb. The observed impairment for the injured limb at RTP was not evident during the preseason (first time point) and it had resolved by the end of the season (third time point). 

Overall, these findings suggest that altered sprinting biomechanics may be evident at the time of RTP following a recent HSI, but such alterations potentially resolve within a certain time frame. Nevertheless, these observations are based on a very limited number of HSI cases, thus any conclusion about longitudinal changes to sprinting biomechanics following HSI remains speculative. Further quality research is required to explore this issue in greater detail.

### 4.4. Summary

No trends emerge from the available cross-sectional analyses. The main findings from the 14 studies contained in Table 3 and Table 4 are almost evenly split: eight studies [38,40,41,42,44,45,49,51] provide data (of some sort) indicating that differences in sprinting biomechanics do exist post HSI, whereas six studies [39,43,46,47,48,50] provide evidence to the contrary. It is also worth noting that for the eight studies that did report significant findings for certain variables, there were still many other variables evaluated that were not found to differ. According to the QUIPS tool, 11 of the 14 studies (∼80%) had a high risk of bias (Table 2), which therefore prevents firm conclusions being drawn. Even when the findings from the three studies with a low risk of bias are considered only, mixed outcomes are still evident.

There are many methodological factors that should be kept in mind when interpreting the findings from the studies listed in Table 3 and Table 4. First, it is possible that some outcome measures may be more sensitive to hamstring function during sprinting than others. Second, studies adopting a within-participant between-limb design (Table 3) rely on the assumption that lower-limb biomechanics during sprinting should be symmetrical. While this assumption may be valid, there is also some evidence suggesting that interlimb asymmetry in healthy sprinters is more likely to be the norm rather than the exception [59,60,61,62]. It is also possible that unilateral HSI influences the mechanics of both lower-limbs, as has been found to be the case with other injuries [63,64]. Hence, the uninjured lower-limb may not represent an appropriate reference. Third, although recruiting a large number of well-matched participants can be difficult, the sample sizes used in the majority of studies have been relatively small and could be subject to type 2 statistical errors (i.e., negative findings may represent false negatives). Equally so, the lack of preregistration together with the typically large number of variables examined may inflate the probability of type 1 errors (i.e., positive findings might be capitalising on chance). Fourth, details about the rehabilitation protocol and training regimen implemented following HSI (which likely has a significant influence on the outcome) are unfortunately difficult to obtain from retrospective recall, hence this information is usually not available. Fifth, HSI classification (i.e., injury definition, location, mechanism of injury, severity, time of occurrence) has not been done in a systematic way across the various studies. Sixth, most studies have used self-report measures to collect injury data, so they are prone to recall bias. Seventh, the time of HSI occurrence used in the studies presented in Table 3 and Table 4 varies from two months to ~5 years prior to study recruitment. The findings from two studies monitoring change over time for a limited number of HSI cases suggest that alterations to sprinting biomechanics evident at RTP may not persist indefinitely [39,58]. Therefore, analysing data from a small number of participants with large differences in the time between the prior HSI and study recruitment could be problematic. Eighth, the mechanism of injury is rarely reported, thus the previously injured cohorts likely included people who sustained HSIs in a variety of different ways. This heterogeneity could be an issue if a running-related HSI affects sprinting biomechanics differently to a kicking- or stretch-related mechanism of injury [65]. Ninth, in some studies, the inclusion criteria only required the participants to miss a minimum of 48 h of training and/or match exposure, which raises some concerns about HSI severity. According to the Fuller et al. [66] consensus statement on injury severity classification, this time period corresponds to minimal and mild severity injuries. It is possible that sprinting biomechanics variables are less likely to be affected after a minor HSI compared to a more severe injury. Overall, the current state of play from the cross-sectional studies listed in Table 3 and Table 4 should be interpreted as an absence of evidence rather than evidence of absence. There are not enough high-quality studies available to confidently establish if and how prior HSI affects sprinting biomechanics.

## 5. Could Sprinting Biomechanics Be a Risk Factor for Hamstring Strain Injuries?

The major limitation of the studies listed in Table 3 and Table 4 is that they provide no information about cause or effect. It is not known whether any differences in sprinting biomechanics variables (if observed) existed prior to the injury and could be causative factors, or whether they were merely a consequence of the injury and thus should be considered unresolved impairments. To address this issue, prospective studies investigating the association between sprinting biomechanics and future HSIs need to be considered. To our knowledge, only five such studies have been published to date (Table 5) [47,48,51,52,53].

One prospective study focused on trunk and hip muscle sEMG activity during over-ground sprinting. Schuermans et al. [52] recorded sEMG activity from the external and internal obliques, erector spinae, gluteus maximus, medial hamstrings and BF for 51 amateur soccer players. Participants maximally accelerated over 40 m on an indoor track and experimental data were captured between 15 and 25 m from the starting location. Participants were monitored for 18 months following baseline testing. Using statistical parametric mapping to analyse the data, players who did not experience a HSI (n = 36) displayed significantly higher normalised sEMG activity for the gluteus maximus during late swing than players who did not sustain an injury (n = 15). Uninjured players also displayed significantly higher normalised sEMG activity for a cluster of trunk muscles (combined sEMG activity for the external and internal obliques and erector spinae) during early swing. Using binary logistic regression, the risk of sustaining a HSI was reduced by 20% for each 10% increment in gluteus maximus sEMG activity during late swing (*p* = 0.023; odds ratio, 0.98; 95% CI, 0.963–0.997) and by 6% for each 10% increment in the trunk muscle cluster sEMG activity during early swing (*p* = 0.007; odds ratio, 0.99; 95% CI, 0.989–0.998). It was concluded that higher trunk and gluteal muscle activity during the swing phase of sprinting may be important for reducing the risk of HSI.

The other four prospective studies focused on kinematic and/or kinetic variables during over-ground sprinting [47,48,51,53]. Haugen et al. [48] examined 14 different kinematic variables relating to interlimb asymmetry during sprinting, but none were significantly different between athletes who suffered a HSI within a 12 month follow-up period (n = 12) and those who did not (n = 9). In contrast, Schuermans and colleagues [47] collected trunk and lower-limb kinematic data during sprinting for 29 amateur soccer players. Four players went on to suffer a HSI during the 1.5 season follow-up period. Compared to matched controls, the subsequently injured players displayed significantly greater anterior pelvic tilt during early swing and significantly greater thoracic lateral flexion towards the ipsilateral side during late swing. Kenneally-Dabrowski and colleagues [53] recorded trunk kinematics as well as lower-limb kinematics and kinetics during sprinting for 10 professional Rugby Union players. Participants maximally accelerated over 50 m on an indoor track and data were captured between 30 and 50 m from the starting location. Data were analysed for the swing phase only and players were monitored over the entire competition season following baseline testing. Using functional component analysis to identify patterns of variability in the kinematic and kinetic data, subsequently injured players (n = 3) were found to display increased thoracic lateral flexion towards the ipsilateral side as well as a greater peak hip extension moment and increased peak knee joint power absorption during late swing [53]. However, no difference between groups was found for the degree of anterior pelvic tilt during sprinting. Most recently, Edouard et al. [51] analysed the association between sprint acceleration mechanics and the occurrence of HSIs in a cohort of 284 soccer players. The radar gun system was used to capture data of interest: the predicted maximal horizontal force production at zero velocity and the predicted maximal sprinting velocity where horizontal force can still be produced. Players were tested at various timepoints throughout the season, with the number of tests completed per participant ranging from one to six. A total of 47 new HSIs were observed in 38 players. Whilst baseline data were not found to be associated with new HSI occurrences, when data collected at all timepoints throughout the season were considered, a significant relationship was revealed between lower predicted maximal horizontal force production and a higher likelihood for a new HSI occurring within the weeks following testing. 

The QUIPS tool found all five prospective studies to have a high risk of bias (Table 2), thereby limiting our ability to draw any firm conclusions when collating findings. These studies also have some other limitations worth noting. First, to make between-muscle comparisons with respect to the amplitude of sEMG activity, Schuermans et al. [52] used separate isometric maximum voluntary contractions to normalise the data. Whilst this approach is included in the Surface Electromyography for the Non-Invasive Assessment of Muscles (SENIAM) guidelines [67], the conditions differ dramatically from the task of interest (i.e., sprinting). Ultimately, between-muscle comparisons of signal amplitude for sEMG activity are critically dependent upon the particular method used to normalise the data, hence the findings from Schuermans et al. [52] must be interpreted with this point kept in mind. For example, gluteus maximus normalised sEMG activity during late swing and early stance for players who did not experience a HSI had an average amplitude between 200% and 300%, which would indicate that the normalisation task for gluteus maximus sEMG activity in this study did not elicit true maximal voluntary contractions. Second, Kenneally-Dabrowski et al. [53] used inverse dynamics to compute joint moments and powers, but data were expressed in absolute units, therefore it is possible that the reported differences in joint kinetics may be attributable to variability in anthropometric properties between subjects. Kenneally-Dabrowski et al. [53] also did not quantify sprinting biomechanics during stance, which is a phase in the stride cycle when the hamstrings are known to be highly activated and generating force [32,68] and thought by some to be vulnerable to injury [16,69]. Third, only a relatively small number of HSIs were observed when pooling numbers across these five prospective studies. The total number of participants was n = 395, with n = 72 (18%) of these participants suffering a HSI during the follow-up period. Fourth, none of the studies adequately addressed the effect of confounding variables. For example, one important confounder is exposure to sprinting and other high-intensity training and/or match-play activities throughout the follow-up period. Edouard et al. [51] did capture weekly exposure in hours of football training and competition. However, none of the prospective studies formally quantified (e.g., using GPS data) exposure to relevant high intensity sprinting actions.

## 6. Review Limitations

A formal systematic review was not undertaken, mainly because of the overall low-quality evidence from the relatively small number of available studies plus the heterogeneous protocols used in the studies to record sprinting biomechanics data. Instead, a detailed summary of the relevant research has been provided, which is considered reasonable practice under such circumstances [70].

## 7. Future Directions

Future research should address some of the limitations highlighted in this review. Studies need to be designed according to a quality assessment tool such as the QUIPS and preregistered so that the planned research hypotheses are documented a priori. To explore the effect of prior HSI on sprinting biomechanics, cross-sectional studies can be conducted where homogenous groups are recruited. A control group comprised of athletes that have all had sufficient and regular training and/or match exposure and have never suffered a HSI during their sporting career could be compared to a group of participants with a history of unilateral HSI (e.g., time of injury occurrence between 6–12 months prior to study recruitment). Each HSI case should ideally have radiological confirmation (e.g., MRI), involve the same muscle (e.g., BF_LH_), have a similar mechanism of injury (e.g., running-related), and be of sufficient severity where a significant layoff from full training and/or matches was required (e.g., minimum 3–4 weeks). There may be benefit in repeating cross-sectional analyses at various time points following a HSI to determine the longitudinal behaviour of any observed impairments in sprinting biomechanics. For example, variables of interest could be recorded towards the end of the rehabilitation period when the athlete has recommenced sprinting, at the time of RTP, and then at predefined time points following RTP (e.g., every 2 months) for a certain follow-up period (e.g., 6 months). To decipher cause or effect, large scale prospective studies are fundamental to establish if certain sprinting biomechanics variables are associated with HSI risk, but these studies will always be challenging to undertake [71]. The difficulty of recruiting a cohort of participants with a large enough number of HSI cases (i.e., index and/or recurrent injures) to complete a sufficiently powered prospective study could potentially be overcome by exploring collaborative opportunities involving several research groups with similar interests, an approach that has recently been adopted by Edouard et al. [51].

## 8. Conclusions

This narrative review collated the available evidence underpinning the potential link between sprinting biomechanics and HSIs. Fourteen studies addressed the question of whether prior HSI affects sprinting biomechanics. Because of mixed outcomes and a high risk of bias for 11 of these studies, a definite answer to this question could not be determined. Five studies investigated whether sprinting biomechanics might pose a risk for future HSI. Despite four of these studies reporting some significant associations, they were all assessed as having a high risk of bias. Whilst the studies completed to date have delivered some interesting findings and stimulated some new directions for future research, unfortunately the available evidence was unable to provide a clear and nonconflicting perspective on the potential link between sprinting biomechanics and HSIs.

## Figures and Tables

**Table 1:** Keyword grouping used during the systematic search.

Muscles	Injury	Timing	Running	Biomechanics
Hamstring *	Injur *	Past	Run *	Mechanic *
Semitendinosus	Strain	Prior	Sprint *	Biomechanic *
Semimembranosus	Tear	Retrospectiv *	Acceleration	Kinematic *
‘Biceps Femoris’	Pull	Previous *		Kinetic *
‘Posterior Thigh’	Rupture	Recent *		Techni *
Thigh	Torn	Histor *		
		Prospectiv *		

* Truncation. Boolean term OR was used within groups, while AND was used between groups.

**Table 2:** Risk of bias assessment.

	Potential Risk of Bias Domain						
Retrospective Studies	1	2	3	4	5	6	Risk of Bias
Iboshi et al. [41]	-	+	-	-	-	+	High
Lee et al. [42]	-	+	+	+	-	+	High
Slider et al. [43]	+	-	+	+	-	+	High
Brughelli et al. [44]	+	+	-	+	-	+	High
Mendiguchia et al. [39]	+	+	+	+	+	-	Low
Daly et al. [45]	+	+	-	+	-	+	High
Barreira et al. [46]	+	+	-	+	-	+	High
Schuermans et al. [47]	+	-	-	-	-	+	High
Haugen et al. [48]	-	+	-	+	-	+	High
Higashihara et al. [49]	+	+	-	-	-	+	High
Lord et al. [38]	+	+	+	+	-	+	Low
Crow et al. [50]	+	+	-	-	-	+	High
Ishøi et al. [40]	+	+	+	+	-	+	Low
Edouard et al. [51]	+	-	-	+	-	+	High
Prospective studies							
Schuermans et al. [52]	+	-	-	+	-	+	High
Schuermans et al. [47]	+	-	-	-	-	+	High
Haugen et al. [48]	-	+	-	+	-	+	High
Kenneally-Dabrowski et al. [53]	+	+	-	+	-	+	High
Edouard et al. [51]	+	-	-	+	-	+	High

1, study participation; 2, study attrition; 3, prognostic factor measurement; 4, outcome measurement; 5, study confounding factor; 6, statistical analysis and reporting.

**Table 3:** Cross-sectional studies investigating within-participant between-limb differences in sprinting biomechanics in people with a history of unilateral HSI.

References	Study Population	Injury Occurrence Period	Methods	Tasks	Variables	Results (IL vs. NIL)
Lee et al. [42]	12 males from various running-based sports Hx	1–36 months	Laboratory based. Over-ground running. Data measured using 3D MOCAP combined with a force plate.	6 x submaximal running trials at 80 % of maximum speed (mean = 7.7 ± 0.1 m/s).	- Sagittal plane hip and knee joint θ, ω, M and P for 3 stride cycles (both legs)	- Lower peak hip flexion θ (−1.9°) for IL during swing phase (*p* = 0.02–Cohen’s d = −0.4)
Silder et al. [43]	15 participants (males and females) from various running-based sports Hx	5–13 months	Laboratory based. Motorised treadmill. Data measured using 3D MOCAP, sEMG system (BF, RF, VL and MH) and musculoskeletal modelling.	Running trials at 60, 80, 90 and 100% of maximal sprinting (Mean = 7.6 ±1 m/s)	- Peak BF_LH_ MTU stretch - sEMG onset and offset of RF, VL, LH and MH	- No difference in MTU stretch was found - No difference in sEMG onset and offset across running speeds
Brughelli et al. [44]	11 male semi-professional AFL players Hx	1–24 months	Laboratory based. Non-motorized treadmill. Horizontal force: measured with a nonelastic tether attached to the participant with a harness and connected to a horizontal load cell. Vertical force: measured by 4 load cells mounted under the running surface.	8 s of steady-state running at 80% of maximum speed.	- Vertical and horizontal GRF - Vertical stiffness - Leg stiffness - Centre of mass displacement - Contact time - Impulse - Positive work	- Horizontal GRF was significantly less (45.9% difference) for IL (*p* < 0.01)
Barreira et al. [46]	6 males professional soccer players Hx	1–24 months	Laboratory based. Non-motorized curved treadmill equipped with force transducers located on the frame supporting the belt.	10 s of maximal sprinting (acceleration and steady-state period included).	- Vertical and horizontal GRF	- No significant between-limb difference in vertical and horizontal GRF
Higashihara et al. [49]	10 male college sprinters Hx	2–61 months	Laboratory based. Over-ground sprinting. Data measured using 3D MOCAP, sEMG system (LH and GM) and musculoskeletal modelling.	Maximal sprinting on 100 m track (average speed: 9.39±0.17 m/s).	- Pelvic anterior tilt, hip and knee joint θ and M - MTU length of the BF_LH_ - Normalised sEMG BF and GM	- IL displayed a lower anterior pelvic tilt θ (late stance, *p* = 0.039), a lower hip flexion θ (mid swing, *p* = 0.02), a greater hip flexion θ (late swing, *p* = 0.049), a greater knee flexion angle (mid swing, *p* = 0.02) - Shorter BF_LH_ length (late swing, p= 0.039) for IL - Reduced sEMG activity of BF (late swing) for IL

CS: cross-sectional, Hx: with a history of HSI, IL: injured limb, NIL: uninjured limb, MOCAP: motion capture, θ: angle, ω: angular velocity, M: moment, P: power, VL: Vastus Lateralis, RF: Rectus Femoris, LH: lateral hamstring, MH: medial hamstring and GM: Gluteus Maximus.

**Table 4:** Cross-sectional studies investigating between-group differences in sprinting biomechanics in people with and without a history of HSI.

References	Study Population	Injury Occurrence Period	Methods	Tasks	Variables	Results (Hx vs. H0)
Iboshi et al. [41]	5 male sprinters Hx vs. 7 male sprinters H0	Not provided	Field based. Over-ground sprinting. Data measured using 2D MOCAP + planar link segment modelling.	100 m sprint (only 5th step post start was analysed)	- Location CG in relation to FC - Thigh and leg segment θ - Hip, knee and ankle joint M	Hx group displayed: - Greater horizontal distance from CG to toe at FC. - Smaller stride length - Larger hip extension M during early stance (*p* < 0.05)
Brughelli et al. [44]	Semi-professional Australian Football players: 11 males Hx vs. 11 males H0	1–24 months	Non-motorized treadmill with a nonelastic tether attached to the participant with a harness and connected to a horizontal load cell to measure horizontal force	8 s steady- state running at 80% of maximum speed	- Vertical and horizontal GRF - Vertical stiffness - Leg stiffness - Centre of mass displacement - Contact time - Impulse - Positive work	- Horizontal force significantly greater in non-injured limb of Hx group in comparison to the right (19.2%) and left (20.5 %) leg of the H0 group - Horizontal force significantly reduced in the injured limb of the Hx group in comparison to the right (31.5 %) and left (32.7%) leg of the H0 group
Barreira et al. [46]	Professional soccer players: 6 males Hx vs. 11 males H0	1–24 months	Non-motorized curved treadmill equipped with force transducers located on the frame supporting the belt.	10 seconds of maximal sprinting (acceleration and steady-state period).	- Vertical and horizontal GRF	- No significant between-group differences were found
Daly et al. [45]	Elite hurlers: 9 males Hx vs. 8 males H0	1–24 months	Laboratory based. Motorised treadmill. Data measured using 3D MOCAP, sEMG system (GM, RF, EO, ES and BF).	10 seconds steady-state running at 20 km/h.	- 3D joint θ of the hip, knee and ankle joints - sEMG activity from previously injured BF and from bilateral GM, RF, EO and ES	During the late swing phase, Hx displayed: - Greater between-limb asymmetry in APT θ (*p* = 0.02), hip flexion θ (*p* = 0.01) and medial knee rotation θ (*p* = 0.03) for Hx - Reduction in sEMG ratio of BF/GM (*p* = 0.03), BF/ES (*p* = 0.01), BF/EO (*p* = 0.01) on the ipsilateral side and a reduction in the sEMG ratio of BF/RF (*p* = 0.02) on the contralateral side
Schuermans et al. [47]	Amateur soccer players: 30 males Hx vs. 30 males H0	1–24 months	Laboratory based. Over-ground sprinting. Data measured using 3D MOCAP (camera between 15–25 m).	12 × maximal sprints over 30 m	- 3D joint θ for hip, knee and ankle; 3D segment θ of the pelvis and thorax	- No significant differences were found
Crow et al. [50]	Professional Australian Football players: 7 males Hx vs. 8 males H0	Not provided	Field based. Over-ground sprinting. Data measured using sEMG system (GM, LH and MH).	Graded running protocol over 100 m: acceleration (40 m), steady-state phase (20 m) and deceleration phase (40 m)	- sEMG onset and offset of GM, LH, and MH during the 20m steady-state phase.	- No significant difference in sEMG temporal behaviour for any muscle
Haugen et al. [48]	7 male sprinters Hx vs. 14 male sprinters H0 (10.8 ± 0.22 m/s)	0–12 months	Field based. Over-ground sprinting. Data measured using 3D MOCAP.	3 × 20-m flying sprints preceded by 30–50 m to build up speed.	- Step velocity - Step length - Step rate - Contact time - Aerial time - Touchdown (TD) θ - Interthigh θ - Liftoff (LO) θ - Thigh and knee θ at LO - Maximal thigh flexion - Range of thigh motion - Knee flexion at maximal - Thigh extension - Horizontal ankle velocity	- No significant difference between groups for any of the sprint asymmetry variables
Mendiguchia et al. [39]	Professional soccer players:14 males Hx vs. 14 males H0	Not provided	Field based. Over-ground sprinting. Data measured using radar gun + biomechanical model to estimate mechanical variables	2 × 50-m maximum velocity sprints	- Velocity - Horizontal force - Maximal power	Cohen’s d effect size (90% confidence limit): - Velocity: 0.63 (−0.05;1.30) moderate - Horizontal force: −0.21 (0.90;0.0.48) small - Maximal power: 0.03 (−0.66;0.72) trivial
Lord et al. [38]	Semi-professional Australian Football players: 20 males Hx vs. 20 males H0	1–24 months	Laboratory based. Non-motorized curved treadmill equipped with 4 load cells on the treadmill belt.	10 × 6 s maximum velocity sprints	- Vertical GRF - Horizontal GRF - Contact time - Flight time	- Reduction in horizontal GRF across repeat sprints (−13%) was significantly greater for group Hx
Ishøi et al. [40]	Sub-elite soccer players: 11 males Hx vs. 33 males H0	0–12 months	Field based. Over-ground sprinting. Data measured using a high speed phone camera + phone application specifically designed to estimate sprint mechanical variables.	6 × 30 m sprints	- Maximal horizontal force - Maximal theoretical sprinting velocity - Maximal horizontal power output - Mechanical effectiveness	- No significant difference in horizontal force production (d = 0.51) and maximal power output (d = 0.06) - Significant difference in maximal theoretical sprinting velocity (H0:7.83 ± 0.44 m/s vs. Hx: 8.28 ± 0.90 m/s) and mechanical effectiveness (lower rate of decline in ratio of forces for Hx)
Edouard et al. [51]	224 youth elite, amateur and professional soccer players.	Entire soccer season	Field based. Over-ground sprinting. Data measured using radar gun/laser distance measurement system + biomechanical model to estimate sprint mechanical variables.	2 × 30 m sprints	- Maximal theoretical sprinting velocity - Horizontal force - Maximal power	- Significant difference for maximal theoretical sprinting velocity. H0 (9.0 ± 0.5 m/s) vs. Hx (9.1 ± 0.5 m/s) - No significant difference in net horizontal force production and maximal power

CS: cross-sectional, Hx: with a history of HSI, H0: with no history of HSI, MOCAP: motion capture, θ: angle, ω: angular velocity, M: moment, P: power, APT: anterior pelvic tilt, BF: biceps femoris, ES: erector spinae, EO: external obliques and GM: Gluteus Maximus.

**Table 5:** Prospective studies investigating the association between sprinting biomechanics and HSI risk.

References	Study Population	Follow Up Period	Methods	Tasks	Variables	Number of Hx	Results
Schuermans et al. [52]	51 ♂ amateur soccer players	18 months	Laboratory based. Over-ground sprinting. Data measured using sEMG system.	12 × maximal sprints over 40 m	sEMG of trunk cluster (external and internal obliques, erector spinae), GM, MH and LH.	15 Hx	H0 displayed: → Significantly higher sEMG of the trunk cluster during early swing (*p* = 0.027). → Significantly higher sEMG of the GM during the late swing phase (*p* = 0.042).
Schuermans et al. [47]	29 ♂️ amateur soccer players	18 months	Laboratory based. Over-ground sprinting. Data measured using 3D MOCAP.	12 × maximal sprints over 40 m	3D joint θ of the hip, knee and ankle joints; 3D segment θ of the pelvis and thorax.	4 Hx	Hx displayed: → Significantly greater APT angle during early swing phase. → Significantly greater thoracic lateral flexion during late swing phase.
Haugen et al. [48]	21 ♂️ sprinters	12 months	Field based. Over-ground sprinting. Data measured using 3D MOCAP.	3 × 20 m flying sprints preceded by 30–50 m to build up speed.	Step velocity, step length, step rate, contact time, aerial time, touchdown (TD) θ, interthigh θ, liftoff (LO) θ, thigh and knee θ at LO, maximal thigh flexion, range of thigh motion, knee flexion at maximal thigh extension and horizontal ankle velocity.	12 Hx	No significant difference reported.
Kenneally-Dabrowski [53]	10 ♂ elite rugby players	Super Rugby season	Laboratory based. Over-ground sprinting. Data measured using 3D MOCAP and Force plates.	3 × maximal sprints over 50 m.	3D joint θ of the hip and knee; 3D segment θ of the pelvis and thorax; 3D hip and knee joint M and P, during the late swing phase.	3 Hx	Hx displayed: → Significantly greater thoracic lateral flexion. → Significantly greater hip joint extension moment. → Significantly greater knee joint power absorption.
Edouard et al. [51]	284 ♂️ youth elite, amateur and professional soccer players.	Entire soccer season	Field based. Over-ground sprinting. Data measured using radar gun/laser distance measurement system+biomechanical model to estimate sprints mechanical variables.	2 × 30 m sprints	- Velocity - Horizontal force - Maximal power	47 injuries in 38 Hx	→ No significant association between Hx and kinetics data when only considering baseline data. → Significant association between Hx and lower net horizontal force production when considering value at each measurement session.

♂: male, Hx: participants suffering HSI, H0: participants who did not suffer HSI, MOCAP: motion capture, θ: angle, ω: Angular velocity, M: moment, P: power, LH: lateral hamstring, MH: medial hamstring and GM: Gluteus Maximus

## Appendix A

**Table A1:** Risk of bias assessment tool (Modified QUIPS).

Biases	Issues to Consider for Kudging Overall Rating of “Risk of Bias”	Judgement	
**1. Study Participation**	**Goal: To judge the risk of selection bias**	**YES**	**NO**
Source of target population	The source population or population of interest is adequately described for key characteristics		
Method used to identify problem	The sampling frame and recruitment are adequately described, possibly including methods to identify the sample, place of recruitment, and period of recruitment		
Inclusion and exclusion criteria	Inclusion and exclusion criteria are adequately described		
Adequate study participation	There is adequate participation in the study by eligible individuals		
Baseline characteristics	The baseline study sample is adequately described for key characteristics		
**Summary Study Participation**	**The study sample represents the population of interest on key characteristics, sufficient to limit potential bias of the observed relationship between the prognostic factor and outcome**		
**2. Study Attrition**	**Goal: To just the risk of attrition bias**		
Proportion of baseline sample available for analysis	Response rate is adequate and is >80%		
Attempts to collect information on participants who dropped out	Attempts to collect information on participants who dropped out of the study are described		
Reasons and potential impact of subjects lost to follow up	Reasons for loss to follow up are described		
Outcome and prognostic factor	Participants lost to follow up are adequately described for key characteristics		
information on those lost to follow up	There are no important differences between key characteristics and outcomes in participants who completed the study and those who did not		
**Summary Study Attrition**	**Loss to follow-up is not associated with key characteristics sufficient to limit potential bias to the observed relationship between the prognostic factor and the outcome**		
**3. Prognostic Factor Measurement**	**Goal: To judge the risk of measurement bias related to how the prognostic factor was measured**		
Definition of the PF	A clear definition or description of the prognostic factors is provided		
Valid and reliable measurement Of PF	Method of prognostic factor measurement is adequately valid and reliable to limit misclassification bias		
	The prognostic factors measured are blinded for outcome measure		
	Continuous variables are reported or appropriate cut-offs are used		
Method and setting of PF measurement	The method and setting of measurement of PF is the same for all study participants		
Proportion of data on PF available for analysis	More than 80% of the study sample has completed data for PF variable		
Method used for missing data	Appropriate methods of imputation are used for missing ’PF’ data		
**PF Measurement Summary**	**PF is adequately measured in study participants to sufficiently limit potential bias**		
**4. Outcome Measurement**	**Goal: To judge the risk of bias related to the measurement of outcome**		
Definition of the Outcome	A clear definition of the Outcome is provided		
Valid and reliable measurement of Outcome	The method of outcome measurement used in valid and reliable to limit misclassification bias		
Method and setting of Outcome Measurement	The method and setting of outcome measurement is the same for all study participants		
**Outcome Measurement Summary**	**Outcome of interest is adequately measured in study participants to sufficiently limit potential bias**		
**5. Study Confounding**	**Goal: To judge the risk of bias due to confounding**		
Important Confounders measured	All important confounders are measured		
Definition of the confounding factor	Clear definitions of the important confounders measured are provided		
Method and setting of Confounding Measurement	The method and setting of confounding measurement are the same for all study participants		
Appropriate accounting for confounding	Important potential confounders are accounted for in the study design		
	Important potential confounders are accounted for in the analysis		
**Study Confounding Summary**	**Important potential confounders are appropriately accounted for, limiting potential bias with respect to the relationship between PF and outcome**		
**6. Statistical Analysis and Reporting**	**Goal: To judge the risk of bias related to the statistical analysis and presentation of results**		
Presentation of analytical strategy	There is sufficient presentation of data to assess the adequacy of the analysis		
Model development strategy	The strategy for model building is appropriate and is based on a conceptual framework or model		
	The selected statistical model is adequate for the design of the study		
Reporting of results	There is a description of the association of the prognostic factor and the outcome, including information about the statistical significance		
	Continuous variables are reported or cut-off points are used		
	There is no selective reporting of results		
**Statistical Analysis and Reporting Summary**	**The statistical analysis is appropriate for the design of the study, limiting potential for presentation of invalid or spurious results**		
